# Imbalanced cortisol/BDNF signals and oxidative stress underscore temporary cognitive impairment in deep divers after in water/surface decompression: proof of concept

**DOI:** 10.1007/s00421-026-06254-1

**Published:** 2026-06-13

**Authors:** Simona Mrakic-Sposta, Simone Di Cianni, Gabriele Lombardi, Enrico Moccia, Gualtiero Meloni, Alessandra Vezzoli, Manuel Marzola, Gerardo Bosco

**Affiliations:** 1 Institute of Clinical Physiology, National Research Council (IFC-CNR), Piazza Dell’Ospedale Maggiore, 3, 20162 Milan, Italy; 2 Institute of Diving Pathophysiology, Italian Navy, Varignano - Le Grazie, 19025 ComSubin, Italy; 3 Department of Medicine and Aging Science, “G. d’Annunzio” University of Chieti-Pescara, 66100 Chieti, Italy

**Keywords:** Oxidative stress, Inflammation, Hormones, Cortisol, BDNF, Decompression, Military

## Abstract

**Background:**

Military deep divers experience extreme environmental stressors, particularly with prolonged diving. Alterations such as hyperbaric conditions and variable O_2_ levels can prompt systemic/local oxidative stress (OxS) and elevated nitric oxide (NO^.^) post-dive release, heightening the risk of inflammation and cardiovascular events. Yet, deep diving (e.g. saturation diving) can also lead to cognitive impairment, whose mechanisms remain largely elusive but relevant to determine.

**Methods:**

Three Deep Divers (Italian Navy-Comsubin), with an average age of 31.00 ± 2.64 years, carried out a technical surface-supplied bounce diving in open water (-89m) in the Mediterranean Sea. From them, we collected saliva and urine samples before diving (T0), after deep diving followed by water-surface decompression (T1), and after complete subemersion (T2). To assess for pro-inflammatory and stress conditions, we evaluated reactive oxygen species (ROS) production, DNA oxidation (8-OHdG), total antioxidant capacity (TAC), nitric oxide (NO^.^) oxidation products, and the levels of interleukin-6, cortisol, brain-derived neurotrophic factor (BDNF), dopamine, and glutamate.

**Results:**

At T1, ROS production increased (+ 121%) and continued to increase just to T2, coupled to a continuous rise in 8-OHdG, and a contemporary decline in TAC. Simultaneously, cortisol levels increased while those of dopamine decreased (-27%). BDNF levels were also sizably elevated (+ 175%). Conversely, no changes in pro-inflammatory markers and NO concentrations were evident.

**Conclusions:**

Oxidative stress and neurotransmitter increase and/or decrease (according to biomarkers evaluated) occur post-deep diving, suggesting preventative strategies to mitigate cognitive impairment.

**Supplementary Information:**

The online version contains supplementary material available at https://doi.org/10.1007/s00421-026-06254-1.

## Introduction

Military divers are subjected to unique environmental stressors, including hyperbaric conditions, variable oxygen levels, prolonged diving, and extreme depths, which can significantly impact physiological processes (Sullivan-Kwantes et al. 2021; Gouin et al. 2025). Surface decompression (Sur-D) is used in military and commercial surface-supplied diving. Initial decompression occurs underwater, with the remainder completed in a deck decompression chamber (DDC). This method improves safety and efficiency (Sullivan-Kwantes et al. 2021; Vann et al 2011; Freiberger et al. 2016; Kirkland et al. 2025; Hexdall et al.^2025^). However, this preventive method may cause cognitive deterioration in some divers, either acutely or after repeated dives (Williams et al. 2022).

Oxidative stress occurs when there is an imbalance between reactive oxygen species (ROS) production and the body’s ability to detoxify these reactive intermediates or repair the resulting cellular damage on lipids, proteins, and DNA, implicated in various pathophysiological conditions (Greenwald 2018; Garcia-Llorens et al. 2025). A study by Eftedal et al. (2013) observed that professional divers exhibited elevated biomarkers of oxidative stress post-dive, indicating increased ROS concentration. In addition, nitric oxide (NO) also plays an important role in the physiological adaptations (Pierini et al. 2015) associated with scuba diving. Increase in NO concentration is reported (Mrakic-Sposta et al. 2020). An increase in exhaled nitric oxide levels and nitric oxide metabolites post-dive suggested elevated NO production during saturation diving. Furthermore, in animal models, extensive simulated diving aggravated endothelial dysfunction, primarily through altered nitric oxide signaling, indicating potential cardiovascular risks (Berenji Ardestani et al. 2020; Gallo et al. 2024).

The physical exertion and environmental conditions associated with diving can elicit inflammatory responses. Zarak et al. (2021) and Arya et al. (2023) reported that divers experienced transient increases in pro-inflammatory cytokines following dives, suggesting an acute inflammatory response. Most recently, Mrakic Sposta et al. (2020) reported that 30 days in Saturation Dive compromise the oxidative balance with a consequent increase in inflammatory status, immune activation by neopterin levels and renal impairment. As documented in the literature (Gieseg et al. 2018), elevated levels of neopterin may be associated with oxidative stress, inflammatory processes, and a broad spectrum of pathological conditions and physiological stressors.

Moreover, subacute and chronic oxidative stress and inflammation status have been implicated in alterations to neuroplasticity, potentially affecting cognitive function. Increase in depths of diving showed a significant trend toward worse attention, concentration, working memory, and verbal memory (Lavoute et al. 2008; Kim et al. 2017; Vezzoli et. al. 2024).

Research by Kjeld Simsek et al. (2011) indicated that repetitive exposure to hyperbaric environments might influence neural adaptability, although the precise mechanisms remain under investigation. Despite that, Bosco et al. (2023) show that also “one shoot” exposure to hyperbaric conditions at − 50 m can have adverse effects on cognitive impairment and oxidative stress. A study examining divers experiencing narcosis—a condition induced by breathing gases at high pressure—found a decline in both dopamine and brain-derived neurotrophic factor (BDNF) levels, correlated with cognitive impairments. The authors suggest that hyperbaric-induced alterations in dopamine and BDNF may underlie some of the cognitive challenges observed in divers. Finally, cortisol, a primary stress hormone, plays a crucial role in the physiological responses to scuba diving; elevations in plasma cortisol levels, indicating activation of the hypothalamic–pituitary–adrenal (HPA) axis in response to the hyperbaric environment were reported (Monnoyer et al. 2021). Furthermore, modification in glutamate levels could reflect changes in neurotransmission, associated with mental fatigue and anxiety (Martin et al. 2009; Mechawar et al. 2016).

For all these reasons, our case report with participants aims to investigate oxidative stress, inflammatory changes, and hormonal imbalances, with potential implications for neuroplasticity in military divers. The goal is to elucidate these relationships and develop strategies to mitigate adverse effects, thereby ensuring the health and operational readiness of military and/or commercial diving personnel.

## Material and methods

### Human subjects enrolled in the study

Three scuba divers belonging to the Italian Navy (Comsubin) carried out a technical surface-supplied bounce diving in open water (− 89 m, 9,9 bar, 28 min) in the Mediterranean Sea. Scuba divers were expert male specialists, with an average age of 31.0 ± 2.64 years: 1st 33aa; 2nd 32aa; 3rd 28aa; average height and weight: 177.6 ± 5.5 cm, 76.0 ± 4.0 kg: 1st 178 cm x 80 kg; 2nd 173 cm x 72 kg; 3rd 182 cm x 76 kg; in good health conditions, medically certified to Marina Militare Italiana, Art 10 (https://concorsi.difesa.it/mm/VFI/2025/Pagine/home.aspx); details in Table 1. All subjects were informed about the aims and provided written informed consent. This study was approved by the Human Ethical Committee (HEC-DSB/04-19) of the Department of Biomedical Science of the University of Padova (Italy) and was carried out in accordance with the standard set by the Declaration of Helsinki (World Medical Association 2013).

**Table 1:** Anthropometric, clinical, and cardiopulmonary characteristics of the scuba divers

	Scuba divers		
Anthropometric data	1	2	3
Ethnicity	Caucasian	Caucasian	Caucasian
Age (yrs)	33	32	28
height (m)	1.78	1.73	1.82
weigh (Kg)	80	72	76
BMI (Kg/m^2^)	25	24	23
Experience (Years since certification)	9 years	7 years	7 years
Spirometry			
FVC L (PRED%)	5.99 (118)	4.78 (100)	5.13 (95)
FEV 1 L (PRED%)	4.36 (103)	4.03 (100)	4.45 (99)
FEV1/FVC L (PRED%)	72.7 (89)	84.2 (103)	86.8 (106)
EEG - Electrical activity	Normal	Normal	Normal
Cardiopulmonary Exercise Testing (CPET)			
VO_2_ max (mL/min/Kg)	39.9	38.4	38.8
Peak Power (W)	290	250	256
Cardiovascular parameters			
PA (mmHg)	120/80	120/75	115/60
FC (bpm)	68	70	66
ECG rhythm	sinus	sinus	sinus
Hematological parameters			
Azotemia (mg/dL)	337	30	37
Hematocrit (%)	43.70	47.50	44.20
Hemoglobin (g/dL)	15.0	16.1	15.0

Data are presented for individual participants. Anthropometric data include ethnicity, age, height, weight, and body mass index (BMI). Experience refers to years since certification. Spirometry parameters include forced vital capacity (FVC), forced expiratory volume in 1 s (FEV₁), and FEV₁/FVC ratio, expressed as absolute values and percentage of predicted (%PRED). Cardiopulmonary exercise testing (CPET) results include maximal oxygen consumption (VO₂ max) and peak power output. Electroencephalogram (EEG) reports results of electrical brain activity. Cardiovascular parameters include blood pressure (PA), heart rate (FC), and ECG rhythm. Hematological values include azotemia, creatinine, hematocrit, and hemoglobin.

### Surface-supplied bounce diving procedure

**Fig. 1:**
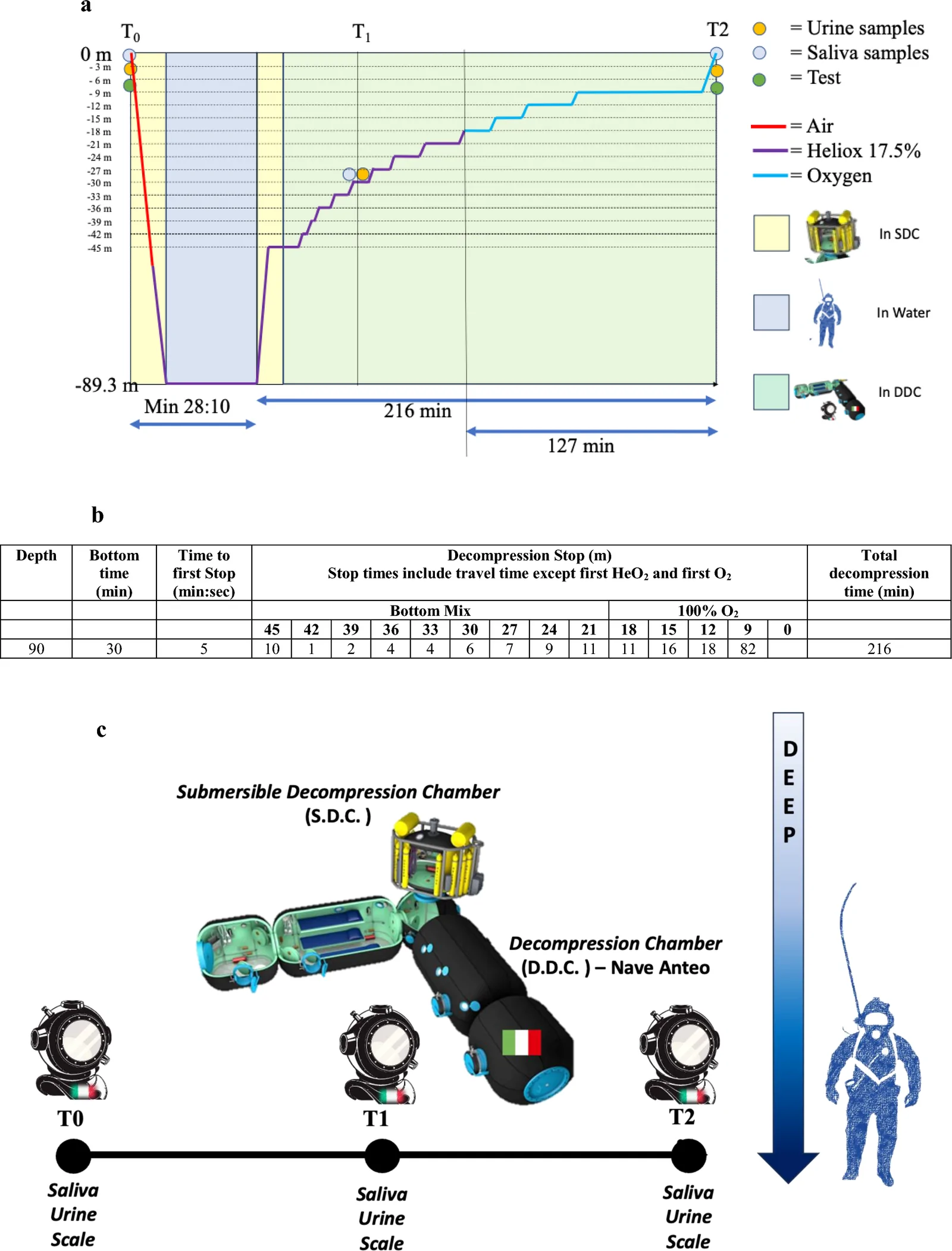
Set-up. **a** Scheme of the decompression profile used and experimental timeline; **b** Dive data: maximum depth 89.3 m; bottom time 28:10 min: sec; descent rate 10 m/min – ascent rate 10 m/min.; **c** Integrated system of Nave Anteo – Marina Militare Italiana (MM) – (DDC); and the Submersible Decompression Chamber (SDC) (modified by https://www.marina.difesa.it/noi-siamo-la-marina/pilastro-operativo/mezzi/forze navali/Pagine/Anteo.aspx)

Surface-supplied bounce diving is conducted by the Italian Navy using an Integrated System installed on board the ship “Anteo”; it is composed of two DDC (Deck Decompression Chamber) and an SDC (Submersible Decompression Chamber) (Fig. 1). Divers enter the SDC (Submersible Decompression Chamber) through the DDC (Deck Decompression Chamber); they wear only wetsuits without helmets and remain at atmospheric pressure. During this phase, it is possible to take the first saliva and urine samples (biological samples) (T0). The SDC is immersed in 85 m but remains at atmospheric pressure inside (external hatch closed). When the SDC reaches 85 m depth, it is possible to start the internal pressurization (dive start). Up to 50 m, the pressurization is done with air; reaching 50 m depth the divers stop and start breathing HELIOX 17.5% Oxygen and 82.5% Helium (bottom breathing gas) through the masks; then the inside pressurization can continue up to 85 m, adding Helium. When the internal (SDC) and external pressure are equal, the external hatch can be opened, and two divers wear helmets and enter the water (wet dive start). The breathing gas is surface-supplied through an umbilical. The third diver (TENDER) remains inside the SDC to support the two divers in releasing and recovering the umbilical and to attend in the eventual recovery of the injured diver within the SDC. At the end of in-water work, the divers return to the SDC, helmets are removed and divers breathe through the masks again.

The SDC is raised to the first decompression stop (both hatches kept open) (the depth stop depends of the dive time). Upon reaching this stop (always 10 min in length), the internal hatch is closed and the SDC is recovered on board in order to perform the mating to the DDC. When the SDC is on board and mated to the DDC, divers transit into DDC and continue the decompression profile up to the surface. They undressed the wetsuit and it is possible to take the second biological samples (T1). Divers’ breath Heliox 17,5% Oxygen up to 18 m and pure Oxygen from 18 to 0 m. At the end of the dive and upon exiting the DDC it is possible to take the third biological sample (T2).

### Body temperature

A digital tympanic thermometer (McKesson LUMEON^™^ - China) measured body temperature pre and post each dive (Purssell et al. 2009; Haugan al. 2013; Dolibog et al. 2022). The tympanic thermometer probe was positioned to fit snugly in the ear canal, following the manufacturer’s recommendations.

### Samples collection

For saliva sampling, the subject was instructed on the correct use of the Salivette device (Sarstedt, Nümbrecht, Germany), as previously reported (Bosco et al. 2023; Mrakic-Sposta et al. 2020, 2024). Salivettes were centrifuged (7 min x 3000 g) and approximately 1 mL of saliva was obtained (Brizzolari et al. 2023). Urine samples were collected via voluntary voiding in a sterile container provided to the subject. At the end of the deep, the samples were transported in ice-dry to the IFC-CNR laboratory in Milan; then all samples were stored at − 80 °C still further processing and analysis.

Saliva and urine samples were collected pre (T0), at deep (T1), and immediately post (T2) dive.

### Biomarkers assessment

Saliva samples were collected to determine: Reactive Oxygen Species (ROS) production, Total Antioxidant Capacity (TAC), brain-derived neurotrophic factor (BDNF), Dopamine (DOPA), Glutamate (GLU), Interleukin-6 (IL-6), and Cortisol concentration; while DNA oxidation (8OH-dG), nitric oxide metabolites (NOx), nitrite (NO_2_), creatinine, neopterin and uric acid were assessed by urine.

### Oxidative stress and antioxidant levels

1) ROS production was measured using an X-band Electron Paramagnetic Resonance (EPR at 9.3 GHz) Spectrometer (e-scan, Bruker, Germany). The Spin probe 1-hydroxy-3-methoxycarbonyl-2,2,5,5tetramethyl-pyrrolidine-hydrochloride (CMH, Noxygen Science Transfer & Diagnostics, Germany) 1 mM was prepared in Krebs-Hepes buffer (KHB) containing 25 μM deferroxamine methanesulfonate salt (DF) chelating agent and 5 μM sodium diethyldithiocarbamate trihydrate (DETC) at pH 7.4. Saliva samples were mixed 1:1 with CMH and analyzed at 37 °C in EPR capillary tubes. Spectra were acquired under identical conditions and converted to absolute concentration values (μmol^•^min^−1^) using the CP• (3-carboxy-2,2,5,5-tetramethyl-1-pyrrolidinyloxy) as external reference. Full methodological details were reported previously (Mrakic-Sposta et al. 2024; Brizzolari et al. 2023; Giacon et al. 2022).

2) Total antioxidant capacity (TAC) was assessed using the Trolox Equivalent Antioxidant Capacity assay (Cayman Chemical, Ann Arbor, MI, USA; Item No. 709001), following the manufacturer’s guidelines and established procedures (Giacon et al. 2025).

3) DNA damage was quantified via immunoassay 8-OH-2-deoxyguanosine (8-OH-dG) EIA kit (Cayman Chemical, Ann Arbor, MI, USA, cat N° 589,320) in urine as previously described (Montorsi et al. 2025). Coefficient of variation (CV) indicated by the manufacturer: inter-assay CV 8.4%; intra-assay CV 6.2%. Absorbance was measured at 412 nm and normalized to creatinine values.

### Inflammatory status

Interleukin IL-6 levels were measured using a human interleukin ELISA kit (Cayman Chemical, Ann Arbor, MI, USA, Item No. 501030), according to the manufacturers’ instructions (Mrakic-Sposta et al. 2024). Coefficient of variation (CV) indicated by the manufacturer: inter-assay range CV 5.40 - 26.22%; intra-assay range CV 4.12—9.28%.

### Nitric oxide metabolites

Nitric Oxide metabolites (NOx = (NO_2_ −) + (NO_3_ −)) level determination was performed by the spectrophotometric method using to Griess reagent in urine, utilizing a commercial colorimetric assay kit (Cayman Chemical, Ann Arbor, MI, USA, cat N° 780,001) as previously described (Green, 1982; Mrakic-Sposta et al. 2024). Samples and standards were read in duplicate at a wavelength of 540 nm. Coefficient of variation (CV) indicated by the manufacturer: inter-assay CV 3.4%; intra-assay CV 2.7%.

### Neuroplasticity biomarkers

Dopamine, BDNF and Glutamate (GLU) concentrations were assessed by ELISA kits according to the manufacturers’ instructions (Cat. No. EU0392, Fine Test, Wuhan, China; Human BDNF ELISA kit, Abcam, USA; Glutamate ELISA KIT, LDS, Germany) (Demartini et al. 2023; Bosco et al. 2025). Samples and standards were read spectrophotometrically at a wavelength of 450 nm.

### Hormone

Salivary cortisol levels were measured using enzyme-linked immunosorbent assay kits (ELISA kit cat. No.: EH0641 FineTest, Wuhan, China), following the manufacturer’s instructions (Bosco et al. 2025; Montorsi et al. 2025). The inter-assay coefficient of variation was in the range indicated by the manufacturer: cortisol < 10%.

### Creatinine, neopterin and uric acid concentration

Urinary creatinine, neopterin, and uric acid concentrations were measured using high-pressure liquid chromatography (HPLC), as previously described (Giacon et al. 2022, 2025). Analytic separations were performed at 50 °C on a 5 μm Discovery C18 analytical column (250 × 4.6 mm I.D., Supelco, Sigma-Aldrich) at a 0.9 mL• min^−1^ flow rate. The calibration curves were linear over the range of 0.125–1 μmol/L, 3.75–60 mmol/L, and 1.25–10 mmol/L for neopterin, uric acid, and creatinine levels, respectively.

All the ELISA determinations were carried out as previously described and using a microplate reader spectrophotometer (Infinite M200, Tecan Group Ltd., Männedorf, Switzerland). All the assessments were duplicated, and the interassay coefficient of variation was in the range indicated by the kit’s manufacturer.

### Scale for assessment of physical fatigue, and subjective perception

Perceived exertion was evaluated immediately after the dive based on physical sensations, with muscle fatigue assessed using the Borg Rate of Perceived Exertion scale. (RPE) (Borg 1982). Before and after dive (T0 vs T2) participants were asked to rate a set of subjective mood, general wellness (happy/unhappy, rested/tired), general sensation (hot/cold, calm/agitation), and head no-pain/pain. They were evaluated using a 0–100 mm visual analog scale (VAS) to test the subjective perception (Burckhardt et. al. 2003; Hawker et al. 2011; Brizzolari et al. 2023; Montorsi et al. 2025).

### Copying an archimedes spiral

Participants were asked to copy with their dominant arm a spiral template printed in black on an A4 paper. The quantitative value was defined through the length of the drawn Archimedean spiral: L = pi*N*R with N = R/t; where: t = spiral step, N = number of rounds, R = max radius of the spiral. We compared the spiral template length with the length of the drawn spiral (Marceglia et al. 2017).

### Data collection and analysis

Data are presented as mean ± SD. Statistical analyses were performed using (JASP version 0.19.3. 2025 and with GraphPad Prism 10.6.1, GraphPad Software Inc., San Diego, CA, USA; https://www.graphpad.com/) for Mac. Data were compared by ANOVA followed by Tukey multiple comparisons test to further check the significance, for all biomarkers. The Wilcoxon matched-pairs signed rank test was used, and a significant difference was set at p < 0.05. Confidence interval (CI) for the estimated values was calculated. dCohen with 95% CI was used for calculating the size effect. Change Δ% estimation ((pre-value/post-value) × 100) is also reported in the text.

In addition, considering the low number of subjects, we also applied Bayesian statistics to further confirm our findings (Keysers et al. 2020; van Doorn et al. 2021). Bayesian statistic is gaining increasing interest specially to confirm negative findings, being able to discriminate between “absence of evidence” and “evidence of absence”. Since model probability is expressed as the ratio between the ability of the null and alternative hypotheses to explain the data, evidence is quantified on a continuous scale rather than through a fixed cut-off (e.g., p = 0.05), also accounting for sample size. In the Bayesian inference, prior information refers to existing knowledge or plausible assumptions about the expected magnitude and direction of the effects before observing the current data. In the present pilot study, we specified weakly informative priors based on general physiological plausibility, to regularize parameter estimation without imposing strong assumptions.

The Bayes Factor (BF) is the ratio between the likelihood of a model including one or more factors and that of the null model (Keysers et al. 2020). Value of BF > 3 is considered as “moderate” evidence in favour of the non-null model, BF > 10 is considered strong evidence. Similarly, BF < 1/3 and BF < 1/10 are considered moderate and strong evidence for the null model (i.e., evidence of absence). If 1/3 < BF < 3, there is not sufficient evidence and the data are inconclusive.

### Data availability

All raw data that were presented or mentioned in the manuscript are available from the corresponding authors on request.

## Results

### Oxy-inflammation

Deep divers undergoing in water or surface decompression display generalized oxidative stress without evidence in inflammatory state.

**Fig. 2:**
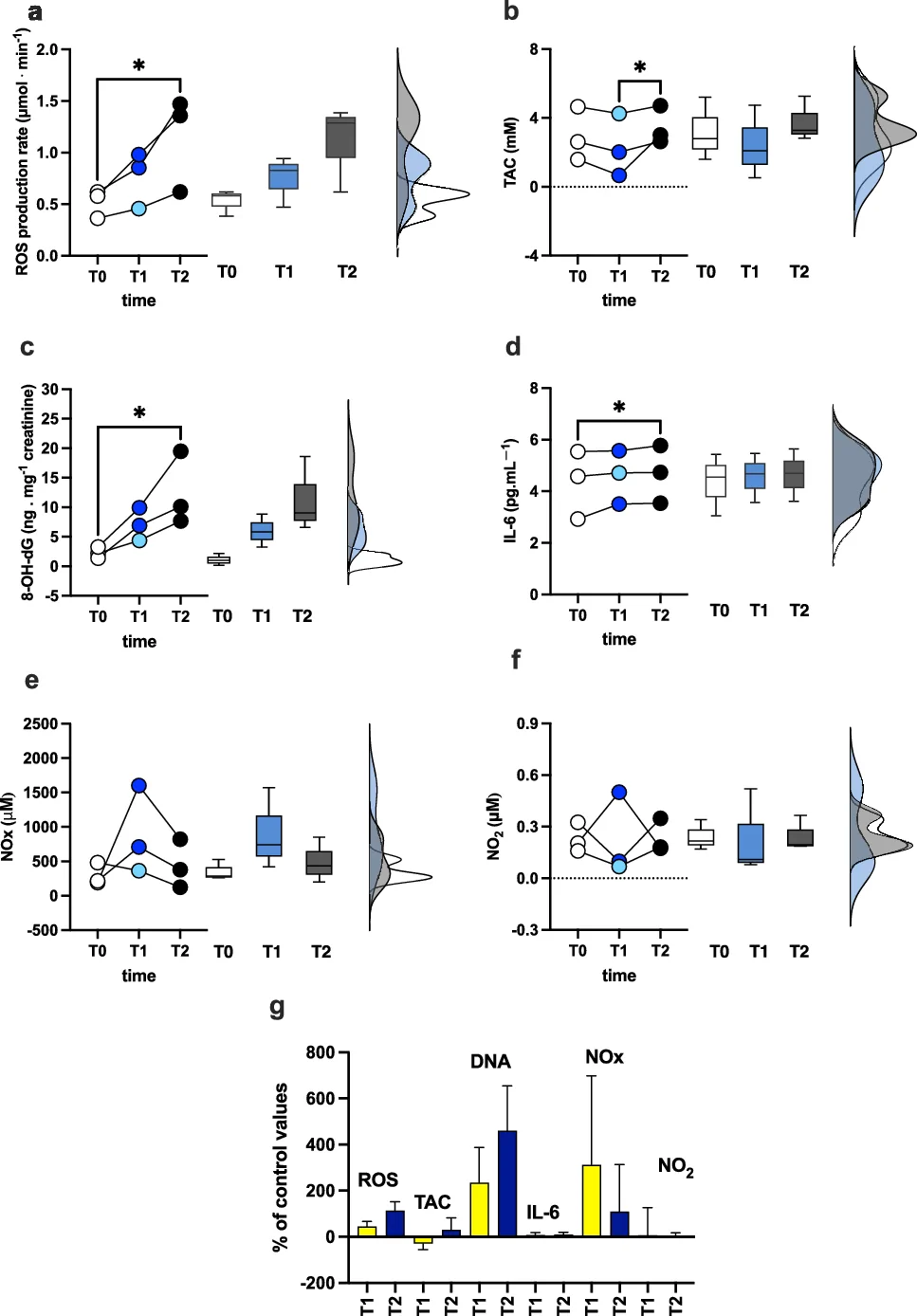
The figure shows the changes of **a** ROS production rate, **b** total antioxidant capacity, **c** DNA damage (8-OH-dG), **d** Interleukin-6 (IL-6), **e** Nitric Oxide metabolites (NOx), (f) Nitrite levels (NO_2_) in divers pre- (T0) during (T2) and post (T2)-dive. Each panel is organized with a graph, on the left, showing line graphs of the trend over time (from T0 to T2) for a single subject: pre- (T0, white) during (T1, in blue two divers and in light blue the tender) and post (T2, grey)-dive; the central panel represents the box plot of the average (mean ± SD) changes observed; and on the right, the corresponding probability distributions. In (G) T1 and T2 values are shown expressed as delta % of pre-exposure (T0) values. Statistically significant differences comparisons are displayed as follows: *, p < 0.05

When comparing pre-diving values (T0) with those obtained at T1, we noticed a progressive rise in ROS production that became significant at T2, i.e., full remersion: (T2 vs T0) 1.150 ± 0.462 vs 0.520 ± 0.137 mmol.min^−1^ (+ 121%; CI 95% − 0.0001–2.29 and 0.18–0.86; dCohen = 1.852; BF = 1.934; Fig. 2a). However, an increase (+ 47%) was recorded at deep too (T1 vs T0) 0.764 ± 0.272 vs 0.520 ± 0.137 mmol.min^−1^ (CI 95% 0.086—1.44 and − 0.0001–2.29; dCohen = 1.115; BF = 1.554). Similar response was recorded on DNA oxidative damage post dive: (T2 vs T0) 12.430 ± 6.228 vs 2.282 ± 0.953 ng.mg^−1^ creatinine (+ 444%; CI 95% − 3.04–27.90 and − 0.086–4.65; dCohen = 2.290; BF = 1.844; Fig. 2c). These data are consistent with a drop in total antioxidant capacity (TAC) occurring at T1 vs T0 2.315 ± 1.813 vs 2.951 ± 1.561 mM (− 21.5%; CI 95% − 2.18–6.81; dCohen = 0.378; BF = 2.484; Fig. 2b) that, however, recovered at T2 3.454 ± 1.104 mM (+ 17%; CI 95% 0.71–6.19; dCohen = 0.370; BF = 0.684). In aggregate, this set of data suggests that deep divers experience post-dive oxidative stress that, however, could be countered by still compensated antioxidant defenses. Despite that, no significant modification at T1 and T2 was recorded in inflammatory status, urinary NO metabolites, and nitrite concentration: IL-6 (T0, T1, T2) 4.346 ± 1.323 vs 4.592 ± 1.039 vs 4.679 ± 1.116 pg.mL^−1^ (Fig. 2d); NOx 296.300 ± 159.500 vs 891.300 ± 638.800 vs 441.500 ± 353.400 µM, and NO_2_ 0.229 ± 0.086 vs 0.222 ± 0.241 vs 0.234 ± 0.098 µM (Fig. 2e, f).

### Neuroplasticity and neuro-excitability

**Fig. 3:**
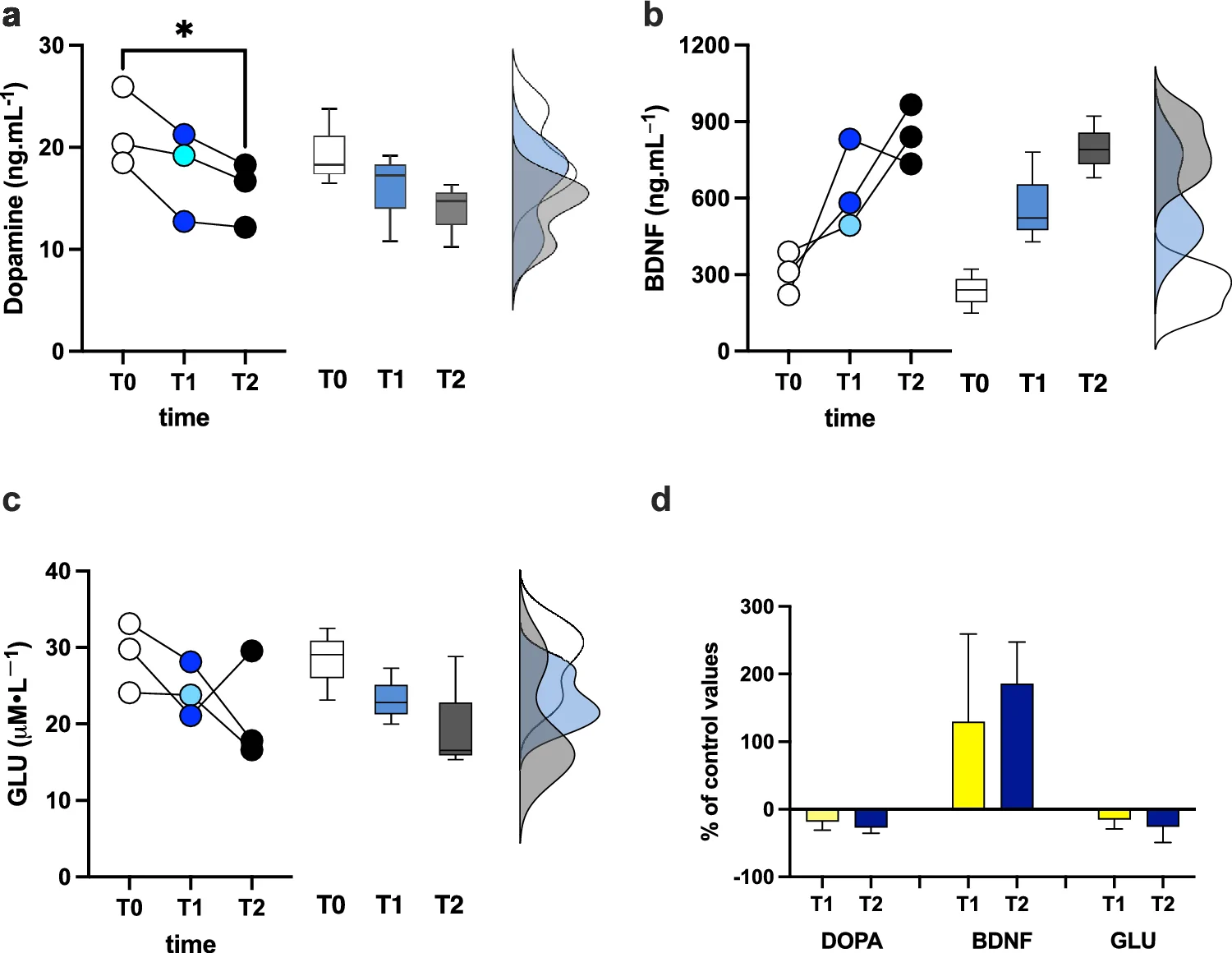
The figure shows the changes of **a** Dopamine, **b** DBNF, and **c** GLU concentration in divers at pre (T0), during (T1) and post-dive (T2). Each panel is organized with a graph, on the left, showing line graphs of the trend over time (from T0 to T2) for a single subject: pre- (T0, white) during (T1, in blue two divers and in light blue the tender) and post (T2, grey)-dive; the central panel represents the box plot of the average (mean ± SD) changes observed; and on the right, the corresponding probability distributions. In **d** T1 and T2 values are shown expressed as delta % of pre-exposure (T0) values of neuroplasticity biomarkers. Statistically significant differences comparisons are displayed as follows: *, p < 0.05

Dopamine and Glutamate levels decline, whereas BDNF increases. Dopamine is a neurotransmitter and plays a role in controlling the movements, as well as emotional responses. Deficiency of dopamine causes slow or effortful movements, known as bradykinesia, postural instability, or problems with coordination and balance, focus, motivation and happiness. Dopamine levels recorded showed a significant decrease during dive (T2 vs T0) 15.710 ± 3.177 vs 21.590 ± 3.870 ng.mL^−1^ (− 27%; CI 95% 7.82–23.61 and 11.97–31.20; dCohen = 1.662; BF = 3.041; Fig. 3a, d).

No significant changes in BDNF concentration were found, but a gradual increase was recorded; while the BF model show a moderate evidence: (T2 vs T0) 847.300 ± 116.300 vs 307.400 ± 83.920 ng.mL^−1^ (+ 175; CI 95% 558.4–1136 and 98.93–515.9; dCohen = 0.9001; BF = 5.689; Fig. 3b, d); similarly GLU level not changed, but a gradual decrease was recorded: (T2 vs T0) 21.330 ± 7.132 vs 29.000 ± 4.564 mM.L^−1^ (-26%; CI 95% 3.61- 39.05 and 17.66–40.34; dCohen = 1.281; BF = 1.003;Fig. 3c, d).

### Cortisol

**Fig. 4:**
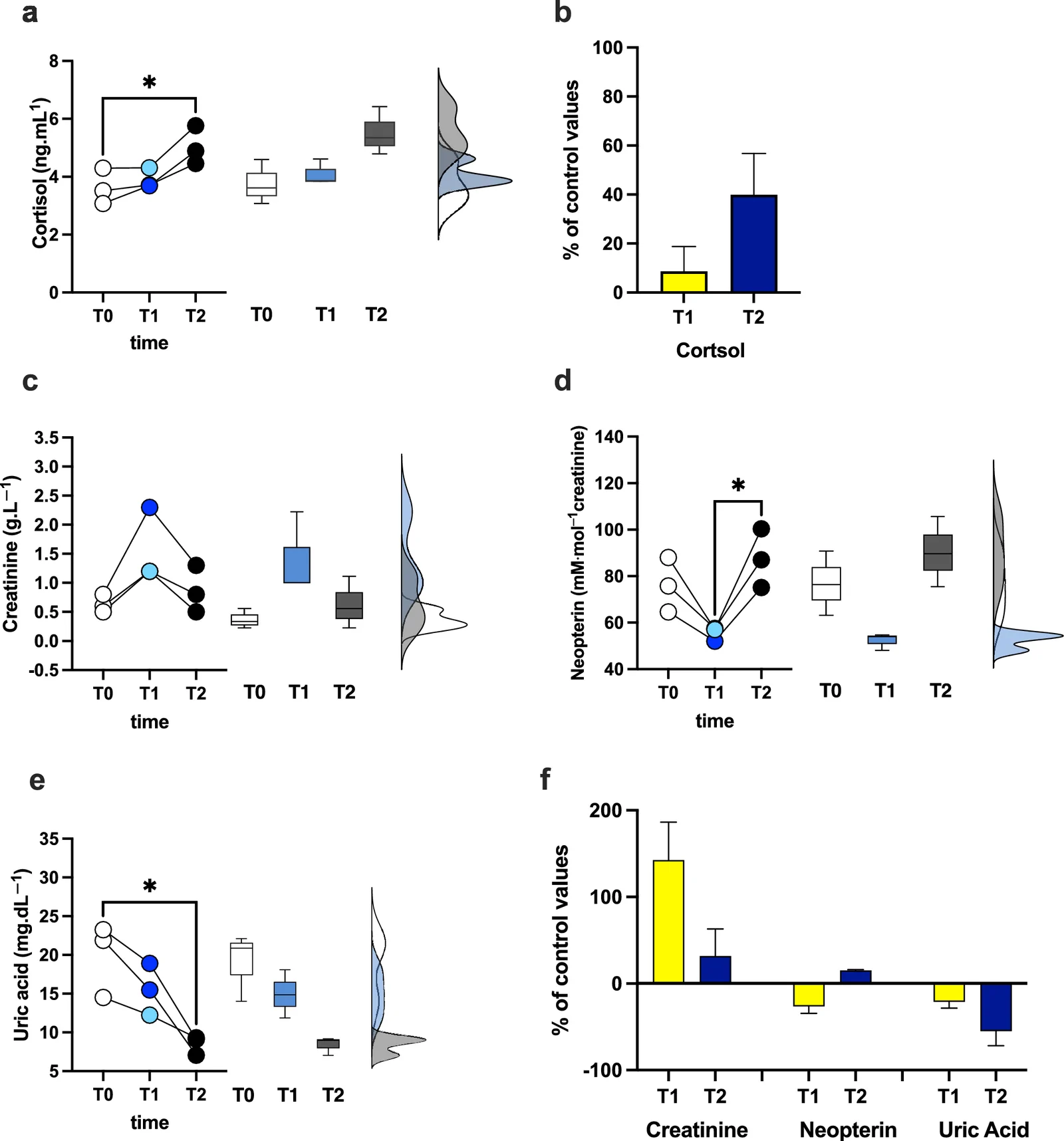
The figure shows the changes of **a** Cortisol concentration, **c** Creatinine, **d** Neopterin, **e** Uric Acid concentration in divers pre (T0), during (T1), and post-dive (T2). Each panel is organized with a graph, on the left, showing line graphs of the trend over time (from T0 to T2) for a single subject: pre- (T0, white) during (T1, in blue two divers and in light blue the tender) and post (T2, grey)-dive; the central panel represents the box plot of the average (mean ± SD) changes observed; and on the right, the corresponding probability distributions. In **b**, **f** a T1 and T2 values are shown expressed as delta% % of pre-exposure (T0) values of stress hormone and renal status biomarkers. Statistically significant differences comparisons are displayed as follows: *, p < 0.05

After water/surface decompression divers display an increase in cortisol hormone and changes in renal status. Cortisol levels went from 3.362 ± 0.618 ng.mL^−1^ (T0) to 5.037 ± 0.663 ng.mL^−1^ (T2), (+ 39%; CI 95% 2.09–5.16 and 3.38–6.68; dCohen = 2.203; BF = 3.750; Fig. 4a, b).

### Renal function

The involvement of renal function is given by the increase of creatinine at deep (T1 vs T0) 1.567 ± 0.635 vs 0.633 ± 0.152 g.L^−1^ (+ 147%; CI 95% − 0.13–1.87 and − 0.01–3.14; dCohen = 2.030; BF = 1.892; Fig. 4c, f), and neopterin at post dive (T2 vs T1) 87.510 ± 12.660 vs 55.530 ± 3.067 mM.mol^−1^creatinine (+ 58%; CI 95% 56.07–118.9 and 47.91–63.15; dCohen = 3.472; BF = 13.629; Fig. 4d, f). A significant decrease in uric acid at (T2 vs T0) 8.500 ± 1.251 vs 19.890 ± 4.705 mg.dL^−1^ (− 57 + %; CI 95% 5.39–11.61 and 8.19–31.57; dCohen = 3.305; BF = 2.142; Fig. 4e, f) was recorded too.

### Physical and subjective scale

**Fig. 5:**
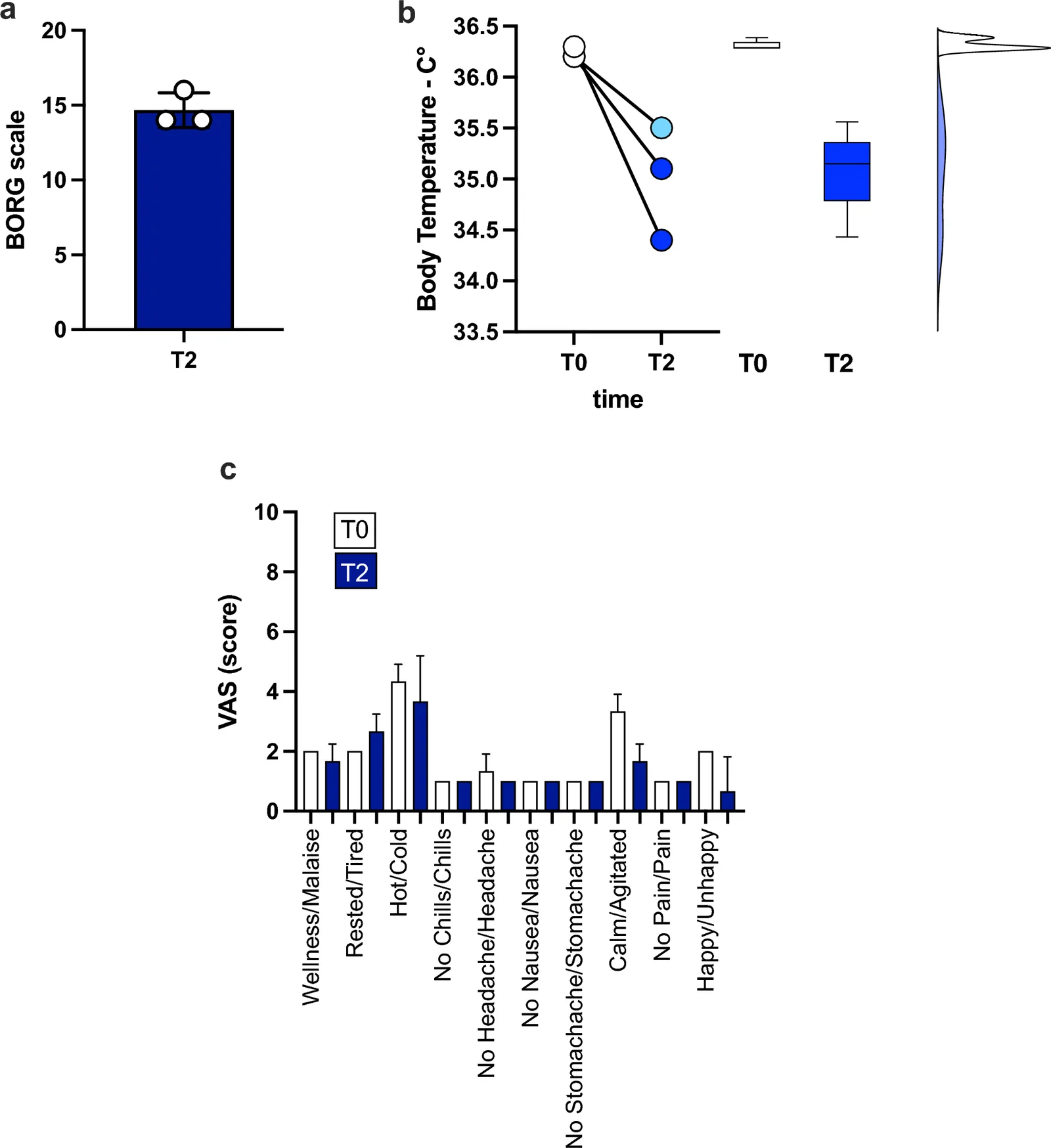
The figure shows the changes of **a** Borg scale post dive, **b** panel of body surface temperature: on the left, showing line graphs of the trend over time (from T0 to T2) for a single subject: pre- (T0, white) during (T1, in blue two divers and in light blue the tender) and post (T2, grey)-dive; the central panel represents the box plot of the average (mean ± SD) changes observed; and on the right, the corresponding probability distributions. In **c** VAS score in divers for general wellness, tiredness, cold, chills, headache, nausea, stomachache, agitation, pain, and happiness were recorded at pre (T0) and post-dive (T2)

Figure 5a reported changes in Borg scores with a mean of 14–16 scores during the deep session. No significant differences are reported in body temperature surface, even if a decrease of 1.23 °C was recorded (BF = 2.032; Fig. 5b). Finally, the VAS item score pre vs post deep session (T0 vs T2) is reported in Fig. 5c. Particularly, we observed percentage changes at T2 compared to T0 in: happiness + 66%; rested/tiredness + 33%; hot/cold -15%; calm/agitated -50%. No pain was recorded. Dives showed a POMS score of 4.6 ± 4.4 at pre dive (T0), reveling vigor and activity.

Finally, to improve the clarity of data interpretation, Table 1 in supplementary material reports a descriptive evaluation for Divers and Tender, including mean ± SD, lower-and upper 95% CI of mean, coefficient of variation in %, and percentage change (Δ%) estimates for T1 vs T0 and T2 vs T0. Given the limited number of participants, we considered it important to also present a descriptive analysis of the data. This approach allows a more transparent representation of data distribution and variability, supporting a more cautious interpretation of the results.

### Divers draw an enlarged copy of an archimedes spiral

**Fig. 6:**
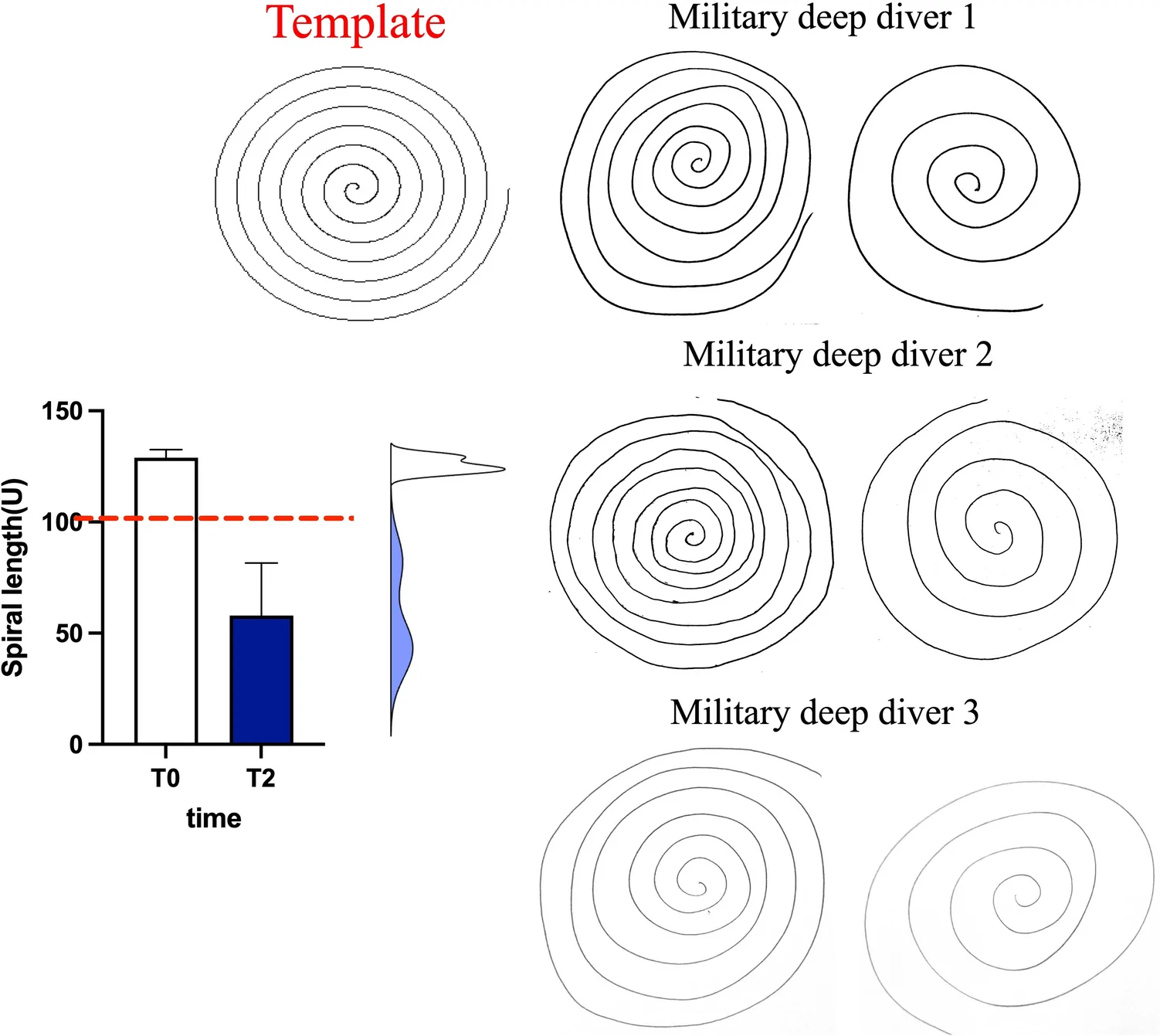
The figure shows the changes of Copy of an Archimedes spiral task. The template of Archimedean Spiral and spiral drawn at pre (T0) and post dive (T2) in all military deep diver. At left the histogram plot of spiral length at pre (T0) and post dive (T2) and the corresponding probability distributions

Qualitative analysis of a Copy of an Archimedes spiral *(*template length 100%; Fig. 6) showed that after deep spiral length decrease (T2 -70%; CI 95% − 0.67–116.6 and 119.7–138.1; dCohen = 4.198; BF = 2.733).

## Discussion

Surface decompression requires a Deck Decompression Chamber (DDC) and is mainly used in professional, military and commercial surface-supplied diving. It allows rapid diver cycling and minimizes in-water decompression in challenging conditions. This technique is used in air and mixed gas (HeO_2_) diving operations (Kirkland 2025). As illustrated previously, it requires specialized operators and technicians; in fact, exceeding the surface interval in Sur-D requires extended chamber decompression, slowing operations. Contaminated water diving increases the interval due to decontamination needs. If DCS occurs, treatment follows established protocols in the DDC (Vann et. al. 2011). While it serves to mitigate decompression sickness, our data suggests that this method may influence oxidative stress levels and cognitive function.

### Oxy-Inflammation in deep divers undergoing water or surface decompression

As widely reported in literature, scuba diving, pressure changes and physical exercise create an imbalance of the reactive oxygen species production, causing damage to the cellular compartments (Brizzolari et al. 2025). As our data show, after compression, 28 min at about 90 m depth, during decompression, scuba diving reported a significant increase in ROS and DNA oxidation, associated with a decrease in antioxidant defenses. These rapid variations could be due to the decompression on the surface, and therefore to the imbalance of the pro-anti-oxidative system. The ROS formation contributes to cell damage and in particular to DNA damage. Cognitive and motor performance may be affected by the stressors associated with surface decompression. Neurocognitive tests post-dive have revealed transient impairments in memory, attention, and executive function (Bosco et al. 2023; Miyake et al. 2000). However, little data are present in the literature on cognitive function, and these deficits could contribute to the formation of oxidative stress impacting neural tissues (Sharma et al. 2023).

### Cognitive function in deep divers undergoing water or surface decompression

The central nervous system (CNS), essential for maintenance of ideal energy levels, crucial for memory, cognition, and mood (Diamond 2013; Lüscher et al. 2012; Reznikov et al. 2009), is critical for adaptation to changes in the environment. Its metabolism regulates extracellular levels, maintaining neural function. Chronic stress and mood disorders impact glutamatergic signaling and neuroplasticity (Pal 2021). A decrease in GLU levels after the bottom phase could be associated with stress factors; in fact, we also recorded an increase in cortisol levels post dive. Physiological and psychological stress, contained during submarine operations, are exacerbated upon return to the surface (Kelly et al. 2025; Sharma et al. 2023; Anegg et al. 2002). Changes in salivary cortisol and urine GLU can be attributed to the combined physiological impact of immersion and changes in environmental pressure (Zarezadeh et al. 2014), associated with work, and/or cold and/or stress and/or mental fatigue (Njire Braticevicet al. 2023; Kelly et al. 2022; Pourhashemi et al. 2016). Based on our data, it seems that 1 h post immersion, brain cognitive function, such as evidenced from the copy of the spiral, changed probably following a decrease in neurotransmitters, which dopamine and GLU. Indeed, dopamine plays a central role in reward-guided learning and motivation (Baik 2020). Stress alters dopamine levels and neuronal activity in the mesolimbic system, affecting coping mechanisms. Dopaminergic modulation helps to adapt behavioral responses to stress (Baik 2020; Pani et al. 2000).

### Physical and subjective scale

After the deep session, participants’ Borg scores reached a mean of 14–16, indicating a substantial perception of physical effort. This occurred alongside a modest decrease in body temperature, averaging 1.23 °C, which may have contributed to the sense of fatigue. This is supported by the VAS, which assessed participants’ subjective responses at T0 and T2. The results indicate that the deep immersion mainly affected fatigue and thermal perception, while pain, gastrointestinal discomfort, and mood were minimally impacted. The generally low post-immersion scores across most categories underscore the participants’ tolerance and adaptation to the deep dive conditions.

### Copying an archimedes spiral

Performance alterations in reproducing an Archimedean spiral after dives persisted for up to 1 h after surfacing (Fig. 6). A decrease in spiral length was observed, expressed in absolute values, albeit with notable inter-individual variability. From the available literature, the use of spiral drawing as a sensitive tool to detect fine motor impairments is well established. Studies in neurological conditions demonstrate that spiral drawing reveals abnormalities in motor control, tremor, scaling, and trajectory deviation (Kim et al. 2020, 2025). The observed variability in scaling (spirals drawn smaller) is consistent with published evidence: spiral drawing tasks reliably capture variability in size and accuracy under conditions of motor or cognitive stress. The fact that subjects could not reproduce a complete spiral (100% model length) but with altered scaling matches the pattern seen in neurological cohorts, where gross task execution remains intact, but fine motor precision deteriorate.

### Limitation and studies in perspectives

One of the limitations of this study is attributable to the very small pool of human subjects qualified to perform dives at such depths, reflecting the highly specialized and exceptional nature of these operations. Second, future studies should include more analyses, such as investigation of specific signaling pathways, ideally supported by well-established animal models under conditions mimicking hyperbaric stress. Third, we acknowledge that the spectrum of hormones and the pro- and anti-inflammatory mediators potentially involved is vast; the current study provides only a preliminary glimpse, which should guide more comprehensive future investigations. Finally, logistical constraints and safety considerations inherent to extreme-depth diving further limit the scope and frequency of data collection in such settings.

## Conclusion

The major findings of this case report with participants do not lie in definitive discoveries, but in providing a valuable starting point for future measurements, while highlight the unique opportunity to study naval personnel operating under recovery operations. The observed increase in oxidative stress and decreases in antioxidant biomarkers can reflect the body’s exposure to high pO₂ and physical exertion during a technical dive. However, it should be noted that the magnitude of these changes varies across biomarkers, with someone showing more pronounced effects than others, which should temper the certainty of the conclusions.

Moreover, evidence indicates that neurotransmitter alterations also occur following deep diving, although the effect sizes are relatively modest. These findings suggest at least two main directions for future research and preventive action. First, the development and application of preventative strategies—such as antioxidant supplementation—could help to avoid or mitigate oxidative stress–related consequences, particularly in professional deep divers who are frequently exposed to high-pressure underwater environments. Second, optimization of decompression procedures might reduce cortisol concentration and dopamine depletion, thereby minimizing the risk of post-diving cognitive impairment.

## Supplementary Information

Below is the link to the electronic supplementary material.

Supplementary file1 (DOCX 35 KB)

## Abbreviations

BDNF: Brain-derived neurotrophic factor
BF: Bayes factor
CMH: 1-Hydroxy-3-methoxy-carbonyl-2,2,5,5-tetramethylpyrrolidine)
CNS: Central nervous system
CP: 3-Carboxy-2,2,5,5-tetramethyl-1-pyrrolidi-nyloxy
DDC: Deck decompression chamber
DOPA: Dopamine
EPR: Electron paramagnetic resonance
GLU: Glutamate
HPLC: High-performance liquid chromatography
IL-6: Interleukin-6
NOx: Nitric oxide metabolites
NO_2_: Nitrite
OxS: Oxidative stress
ROS: Reactive oxygen species
SDC: Submersible decompression chamber
TAC: Total antioxidant capacity
VAS: Visual analog scale
8-iso-PGF2α: 8-isoprostane-PGF2alfa
8-OH-dG: 8-OH-2-deoxyguanosine
