# Supplementary material for: Imbalanced cortisol/BDNF signals and oxidative stress underscore temporary cognitive impairment in deep divers after in water/surface decompression: proof of concept
Source: Eur J Appl Physiol. 2026 Jun 13;126(9):5039–53. doi: 10.1007/s00421-026-06254-1 (PMC13541860; doi:10.1007/s00421-026-06254-1)
Supplement: Supplementary file 1 [file 421_2026_6254_MOESM1_ESM.docx]

**Table 1.** A descriptive evaluation in Divers and Tender.

|  | ***Divers (n=2)*** | | | | | ***Tender (n=1)*** | | | | |
| --- | --- | --- | --- | --- | --- | --- | --- | --- | --- | --- |
|  | **T0**  mean ± SD  [Lower-Upper 95% C.I of mean]  (coefficent of variation %) | **T1**  mean ± SD  [Lower-Upper 95% C.I of mean]  (coefficent of variation %) | **T2**  mean ± SD  [Lower-Upper 95% C.I of mean]  (coefficent of variation %) | **T1 vs T0**  **Δ%** | **T2 vs T0**  **Δ%** | **T0**  Value | **T1**  Value | **T2**  Value | **T1 vs T0**  **Δ%** | **T2 vs T0**  **Δ%** |
| **ROS**  (μmol.min^-1^) | 0.599±0.028  [0.364 0.815]  (4.69) | 0.917±0.089  [0.109 1.725]  (9.79) | 1.414±0.078  [0.706 2.123]  (5.56) | +53 | +136 | 0.364 | 0.457 | 0.619 | +26 | +70 |
| **TAC** (mM) | 2.099±0.726  [-4.42 0.62]  (34.59) | 1.343±0.952  [-7.21 9.90]  (70.95) | 2.825±0.265  [0.44 5.20]  (9.38) | -36 | -35 | 4.653 | 4.258 | 4.710 | -9 | +1 |
| **8-OH-dG**  (ng.mg^-1^ creatinine) | 2.327±1.344  [-9.74 14.40]  (57.75) | 8.415±2.128  [-10.71 27.54]  (25.29) | 14.799±6.619  [-44.67 74.27]  (44.73) | +262 | +536 | 2.191 | 4.387 | 7.684 | +100 | +251 |
| **IL-6**  (pg.mL^-1^) | 4.230±1.849  [-12.39 20.85]  (43.71) | 4.534±1.463  [-8.61 17.68]  (32.27) | 4.653±1.577  [-9.51 18.83]  (33.89) | +7 | +10 | 4.576 | 4.706 | 4.730 | +3 | +3.5 |
| **NOx (**μM) | 204.0±17.39  [48.21 260.80]  (8.50) | 1154.8±632.15  [-4525.00 6834.00]  (54.74) | 600.6±313.10  [-2213.0 3414.0]  (52.13) | +465 | +194 | 480.0 | 364.4 | 123.400 | -24 | -74 |
| **NO_2_ (**μM) | 0.265±0.085  [-0.50 1.03]  (32.33) | 0.299±0.283  [-2.25 2.85]  (94.88) | 0.261±0.123  [-0.84 1.36]  (47.14) | +13 | -1.5 | 0.159 | 0.068 | 0.181 | -57 | +14 |
|  |  |  |  |  |  |  |  |  |  |  |
| **Dopamine** (ng.mL^-1^) | 22.211±5.255  [-25.01 69.43]  (23.66) | 17.006±6.031  [-37.18 71.19]  (35.46) | 15.232±4.336  [-23.73 54.19]  (28.47) | -23 | -31 | 20.338 | 19.222 | 16.677 | -5.48 | -18.00 |
| **BDNF**  (ng.mL^-1^) | 266.402±63.228  [-301.7 83.5]  (23.73) | 706.349±176.589  [-880.2 2293.0]  (25.00) | 850.529±164.243  [-625.1 2326.0]  (19.31) | +165 | +219 | 389.418 | 493.121 | 840.740 | +27 | +116 |
| **Glutamate**  (μM.L^-1^) | 31.451±2.361  [10.23 52.67]  (7.50) | 24.596±4.975  [-20.11 69.30]  (20.23) | 23.683±8.277  [-50.69 89.06]  (34.95) | -22 | -25 | 24.093 | 23.776 | 16.626 | -1.33 | -31 |
|  |  |  |  |  |  |  |  |  |  |  |
| **Cortisol**  (ng.mL^-1^) | 3.298±0.310  [0.57 6.08]  (9.41) | 3.703±0.015  [3.56 3.84]  (0.41) | 4.674±0.308  [1.90 7.44]  (6.59) | +12 | +42 | 4.300 | 4.311 | 5.760 | +0.27 | +34 |
| **Creatinine** (g.L^-1^) | 0.700±0.141  [0.57 1.97]  (20.20) | 1.750±0.777  [-5.23 8.73]  (44.45) | 1.050±0.353  [-2.12 4.22]  (33.67) | +150 | +50 | 0.500 | 1.200 | 0.600 | +140 | +20 |
| **Neopterin**  (mM.mol^-1^creatinine) | 70.205±7.855  [-0.37 140.80]  (11.19) | 54.770±3.917  [19.57 89.97]  (7.15) | 81.065±8.449  [5.14 157.0]  (10.42) | -22 | +15 | 88.030 | 57.050 | 100.390 | -35 | +14 |
| **Uric Acid**  (mg.dL^-1^) | 22.575±0.954  [14.0 31.15]  (4.22) | 17.195±2.425  [-4.59 38.99]  (14.11) | 8.090±1.456  [-4.99 21.18]  (18.01) | -24 | -64 | 14.51 | 12.23 | 9.32 | -16 | -36 |
|  |  |  |  |  |  |  |  |  |  |  |
| **Body Temperature** (C°) | 36.25±0.07  [35.61 36.89]  (0.19) |  | 34.75±0.49  [30.300 39.200]  (1.42) |  | -4 | 36.2 |  | 35.5 |  | -2 |
|  |  |  |  |  |  |  |  |  |  |  |
